# Supplementary material for: A UMLS-based spell checker for natural language processing in vaccine safety
Source: BMC Med Inform Decis Mak. 2007 Feb 12;7:3. doi: 10.1186/1472-6947-7-3 (PMC1805499; doi:10.1186/1472-6947-7-3)
Supplement: Additional file 13 — RAP application directory. Sets up directory for RAP (RDF application for PHP) source code [file 1472-6947-7-3-S13.gz › rap/doc/phpdoc/__filesource/fsource_model__modelResource.php.html]

xml version="1.0" encoding="iso-8859-1"?


File Source for Resource.php


# Source for file Resource.php

Documentation is available at Resource.php

```
1. <?php

3. // ----------------------------------------------------------------------------------
4. // Class: Resource
5. // ----------------------------------------------------------------------------------


9. /**
10. * An RDF resource.
11. * Every RDF resource must have a URIref.
12. * URIrefs are treated as logical constants, i.e. as names which denote something
13. * (the things are called 'resources', but no assumptions are made about the nature of resources.)
14. * Many RDF resources are pieces of vocabulary. They typically have a namespace
15. * and a local name. In this case, a URI is composed as a
16. * concatenation of the namespace and the local name.
17. *
18. * <BR><BR>History:<UL>
19. * <LI>11-09-2003                 : Fixed bug in equals() <radol@gmx.de></LI>
20. * <LI>09-30-2002                : Fixed bug in getNamespace() and getLocalName().</LI>
21. * <LI>09-10-2002                : First version of this class.</LI>
22. * </UL>
23. *
24. * @version  V0.9.1
25. * @author Chris Bizer <chris@bizer.de>
26. *
27. * @package model
28. * @todo nothing
29. * @access    public
30. *
31. */
32. class Resource extends Node {

34. /**
35. * URIref to the resource
36. * @var        string
37. * @access    private
38. */
39. var $uri;


42. /**
43. * Constructor
44. * Takes an URI or a namespace/localname combination
45. *
46. * @param    string    $namespace_or_uri
47. * @param string $localName
48. * @access    public
49. */
50. function Resource($namespace_or_uri , $localName = NULL) {
51. if ($localName == NULL) {
52. $this->uri = $namespace_or_uri;
53. } else {
54. $this->uri = $namespace_or_uri . $localName;
55. }
56. }


59. /**
60. * Returns the URI of the resource.
61. * @return string
62. * @access    public
63. */
64. function getURI() {
65. return $this->uri;
66. }

68. /**
69. * Returns the label of the resource, which is the URI of the resource.
70. * @access    public
71. * @return string
72. */
73. function getLabel() {
74. return $this->getURI();
75. }

77. /**
78. * Returns the namespace of the resource. May return null.
79. * @access    public
80. * @return string
81. */
82. function getNamespace() {
83. // Import Package Utility
84. include_once(RDFAPI_INCLUDE_DIR.PACKAGE_UTILITY);

86. return RDFUtil::guessNamespace($this->uri);
87. }

89. /**
90. * Returns the local name of the resource.
91. * @access    public
92. * @return string
93. */
94. function getLocalName() {
95. // Import Package Utility
96. include_once(RDFAPI_INCLUDE_DIR.PACKAGE_UTILITY);

98. return RDFUtil::guessName($this->uri);
99. }

101. /**
102. * Dumps resource.
103. * @access    public
104. * @return string
105. */
106. function toString() {
107. return 'Resource("' . $this->uri .'")';
108. }

110. /**
111. * Checks if the resource equals another resource.
112. * Two resources are equal, if they have the same URI
113. *
114. * @access    public
115. * @param        object    resource $that
116. * @return    boolean
117. */
118. function equals ($that) {

120. if ($this == $that) {
121. return true;
122. }

124. if (($that == NULL) or !(is_a($that, 'Resource')) or (is_a($that, 'BlankNode'))) {
125. return false;
126. }

128. if ($this->getURI() == $that->getURI()) {
129. return true;
130. }

132. return false;
133. }


136. }

138. ?>
```

Documentation generated on Thu, 7 Jul 2005 13:43:45 +0200 by phpDocumentor 1.3.0RC3
